# Artificially Constructed Quorum-Sensing Circuits Are Used for Subtle Control of Bacterial Population Density

**DOI:** 10.1371/journal.pone.0104578

**Published:** 2014-08-13

**Authors:** Zhaoshou Wang, Xin Wu, Jianghai Peng, Yidan Hu, Baishan Fang, Shiyang Huang

**Affiliations:** 1 Institute of Biochemical Engineering, Department of Chemical and Biochemical Engineering, College of Chemistry and Chemical Engineering, Xiamen University, Xiamen, China; 2 The Key Lab for Chemical Biology of Fujian Province, Xiamen University, Xiamen, China; 3 The Key Lab for Synthetic Biotechnology of Xiamen City, Xiamen University, Xiamen, China; Rochester Institute of Technology, United States of America

## Abstract

*Vibrio fischeri* is a typical quorum-sensing bacterium for which *lux* box, *luxR*, and *luxI* have been identified as the key elements involved in quorum sensing. To decode the quorum-sensing mechanism, an artificially constructed cell–cell communication system has been built. In brief, the system expresses several programmed cell-death BioBricks and quorum-sensing genes driven by the promoters *lux pR* and *P_lacO-1_* in *Escherichia coli* cells. Their transformation and expression was confirmed by gel electrophoresis and sequencing. To evaluate its performance, viable cell numbers at various time periods were investigated. Our results showed that bacteria expressing killer proteins corresponding to ribosome binding site efficiency of 0.07, 0.3, 0.6, or 1.0 successfully sensed each other in a population-dependent manner and communicated with each other to subtly control their population density. This was also validated using a proposed simple mathematical model.

## Introduction

The marine bacterium *Vibrio fischeri* lives symbiotically in light organs of some fishes and squids, and exhibits bioluminescence at high population densities [1]–[3]. Nealson et al. [4] reported that this bioluminescence was due to the accumulation of an autoinducer, and that the intensity of bioluminescence positively correlated with bacterial population density. This phenomenon is called “quorum sensing” [5], and considerable attention has been paid to determine the signaling molecules involved in it. N-3-oxohexanoyl-L-homoserine lactone, a type of acyl-homoserine lactone (AHL), was the first identified molecule in the process [6]. Interestingly, AHL was found to freely diffuse through cell membranes and to play a role in cell–cell communication [7].

As reported, at low bacterial population density, the concentration of AHLs remains low; however, their concentration increases with an increase in bacterial population density. At or above a particular threshold, they effectively bind to LuxR, which has been identified as an intracellular regulatory protein that mediates the formation of the LuxR:AHL complex. This dimer complex subsequently binds to the promoter of *lux* box and induces the expression of downstream genes such as *luxC*, *luxD*, *luxA*, *luxB* and *luxE*, which generates bioluminescence. *V. fischeri lux* box contains the promoters *lux pL* and *lux pR* that regulate the transcription of *luxR* and *luxI*, respectively.

*luxI* encodes a synthase, LuxI, which produces signaling molecule AHLs. *luxI* expression is upregulated by the interaction between the LuxR:AHL complex and *lux* box. Therefore, a positive feedback is involved in sensing signals [8]. In brief, quorum sensing in bacterial bioluminescence is tightly controlled by several key elements such as *lux* box, *luxR*, and *luxI*. This provides the basis for building an artificial quorum-sensing circuit.

You et al. [9] reported a programmed cell-death circuit by cloning *luxR* and *luxI* under the control of a synthetic promoter *P_lac/ara-1_*, and cotransforming the bacterial lethal gene *ccdB* into *Escherichia coli* cells. These engineered bacteria could successfully produce and release autoinducer AHLs, which mediated *luxR* and *ccdB* expression in a feedback manner. As these engineered bacteria grew, they continuously released autoinducer AHLs into their environment. When AHL concentration reached a particular threshold, AHLs combined with LuxR and activated *ccdB* expression to produce CcdB. Consequently, CcdB poisoned bacterial gyrase and resulted in the death of numerous bacterial cells [10]–[12]. However, when bacterial population density decreased, the concentration of LuxR:AHL dimer complexes required to activate *lux pR* was insufficient, and resulted in termination of cell death. Thereafter, the cells entered into a new round of growth. Once population density reached the abovementioned threshold, another round of cell death was triggered. This system successfully mimicked the situation of quorum sensing, in which bacterial cell growth and death could be regulated through bacterial population density.

However, this system was cotransformed using two separate plasmids under the control the promoters *P_lac/ara-1_* and *P_luxI_*. This could be problematic because of an imbalance in the copy numbers of the two plasmids, making it difficult to accurately investigate cellular communication on a genetic basis. In addition, it is difficult to quantitatively manipulate population density at different levels.

Synthetic biology combines biology with engineering to design and build novel biomolecular components, networks, and pathways. It is well known that the features important for synthetic biology include modularity, standardization, and rigorously predictive models [13]–[18]. In recent years, an increasing number of studies in synthetic biology have focused on constructing simple synthetic gene circuits that exhibit desired properties. Meanwhile, a large variety of functional genetic parts are characterized and assembled to construct biological circuits to program new biological behaviors, dynamics, and logical control [19]–[21]. Standard biological parts, such as BioBricks from the “Registry of Standard Biological Parts” (RSBP) at MIT, USA (http://partsregistry.org), provide the foundation for designing and constructing synthetic biological systems [22]. Therefore, a synthetic biological system can be achieved using synthetic biological methods and BioBricks for which gene expression can be precisely regulated and quantitatively and accurately measured [23].

Ribosome binding site (RBS) is a sequence where translation of mRNA into protein starts with. RBS efficiency can be different with binding strengths of various sequences with a ribosome, as a result, yields of protein will be different [24].

In this study, we investigated a few RBSs with different binding strengths (*RBS_0.07_*, *RBS_0.3_*, *RBS_0.6_*, and *RBS_1.0_*). Our aim was to combine the assembly of BioBricks and a bacterial quorum-sensing mechanism to build synthetic circuits to subtly control bacterial population density at different levels.

## Materials and Methods

### Ethics statement

Our research did not involve human subjects; thus, no ethics statement is required.

### Strains and plasmids

The *E. coli* DH5α and BL21(DE3) strains were preserved in our laboratory. The plasmids used in this study included *pSB1A2-P_lacO-1_*, *pSB1A2-lux pL-RBS_1.0_-luxR-TT-lux pR*, *pSB1A2-RBS_0.07_*, *pSB1A2-RBS_0.3_*, *pSB1A2-RBS_0.6_*, *pSB1A2-RBS_1.0_*, *pSB1AK3-RBS_1.0_- luxI-TT*, *pSB1A3-lacZα-ccdB*, and *pSB1AK3-TT*, and were constructed in our laboratory based on the BioBrick plasmids from RSBP with the BioBrick standard assembly method [25], [26].

### Polymerase Chain Reaction (PCR) primers

Primers were designed based on the sequences of target genes. The sequences for five restrictive enzymes (*Eco*RI, *Xba*I, *Pst*I, *Not*I and *Spe*I) and *RBS_1.0_* were introduced at the end of the individual primers for subcloning. The primers were purchased from Shanghai Boya Biotechnology Co., Ltd. and had the following sequences:

2621-F: 5′-GCTCTAGAGAAAGAGGAGAAATACT-3′

2621-R: 5′-AAAACTGCAGCGGCCGCTACTA-3′

### Construction of BioBrick plasmids

To construct BioBrick plasmids, inserts were amplified with 2621-F and 2621-R primers at 94°C for 5 min, followed by 30 cycles at 94°C for 45 s, 59.3°C for 45 s, and 72°C for 1 min, with a final extension at 72°C for 10 min. PCR products were purified using PCR purification kits, followed by digestion with the restriction enzymes *Eco*RI and *Pst*I, ligation with a plasmid vector, transformation into competent cells, and culture on agar plates. Positive clones were confirmed by restriction enzyme digestion and verified by Sanger sequencing. *Taq* polymerase, restriction enzymes, and other related reagents were purchased from TaKaRa Biotechnology (Dalian) Co., Ltd.

### Growth kinetic analysis

Bacteria transformed with plasmids to express *RBS_0.07_*, *RBS_0.3_*, *RBS_0.6_*, or *RBS_1.0_* artificial quorum-sensing circuits were grown in standard Luria-Bertani medium. To assess their growth kinetics, cultures were incubated at 37°C until their optical density at 600 nm reached 0.6–0.8. To induce the expression of inserts, isopropyl β-D-1-thiogalactopyranoside (IPTG) was added at a final concentration of 1 mM. Sample cultures were taken at sequential time intervals, diluted for plating onto agar plates, and incubated overnight at 37°C. Next, the number of clones was counted. Each experiment was repeated at least three times.

### Mathematical modeling

Mathematical modeling has been shown to play an important role in predicting how well a network works, and can be used to optimize the design of synthetic biological models, thereby eliminating the need for a large amount of experimental work [27]–[32]. For this study, we used a mathematical model developed by You et al. [9] that has been validated by experiments and successfully applied to monitor the associations among population density, killer protein concentration and AHL signals. Based on this model, we sought to develop a model to predict the transient dynamics of our synthetic organisms that incorporated artificial population- control circuits.

## Results

### Recombination of BioBrick components

**Figure 1:**
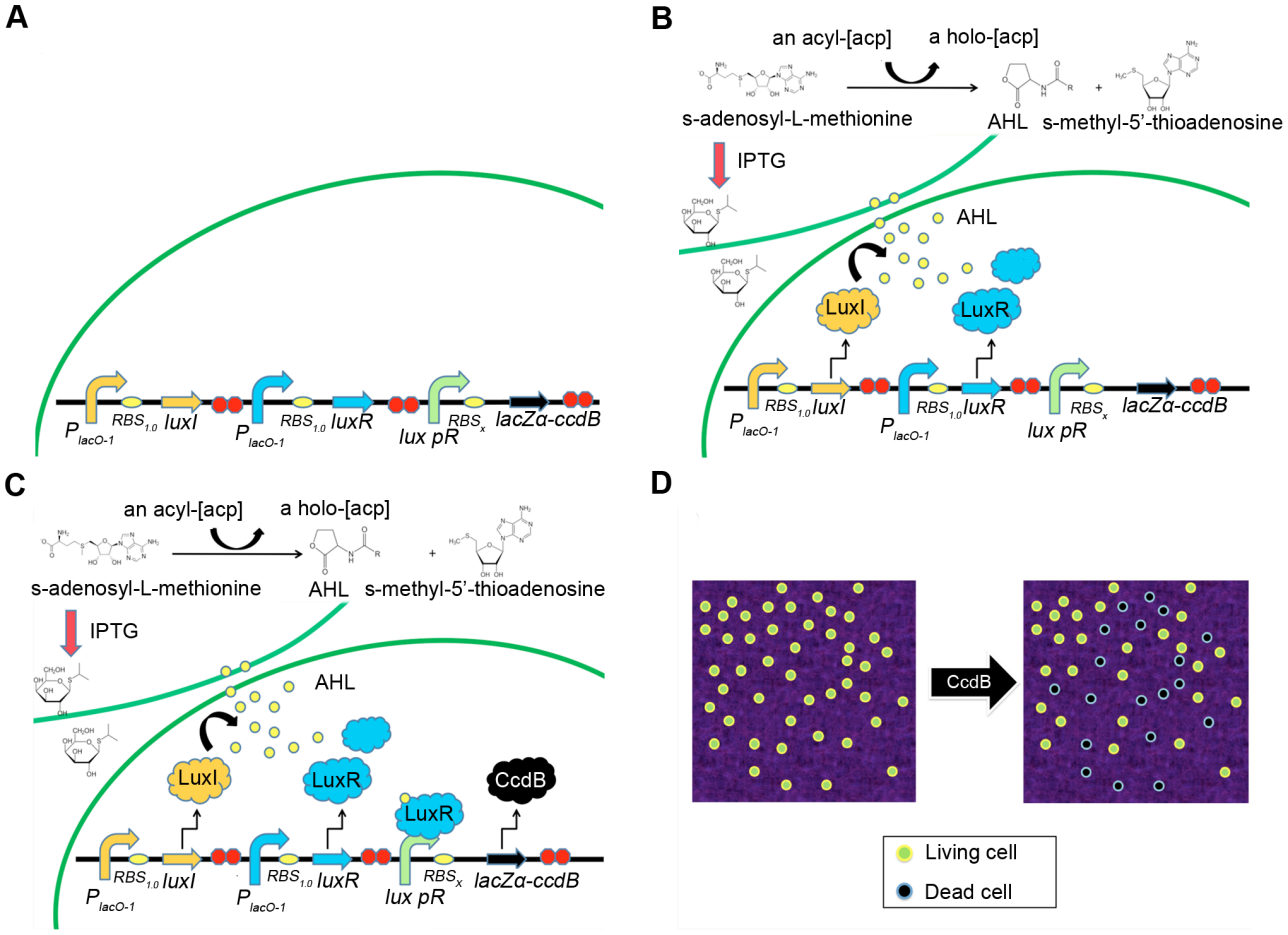
Essential components and mechanism of an artificial quorum-sensing circuit. Note: the subscript “x” in *RBS_x_* represents 0.07, 0.3, 0.6, or 1.0. (A) The essential components of an artificial quorum-sensing circuit are *P_lacO-1_-RBS_1.0_-luxI- TT-P_lacO-1_-RBS_1.0_-luxR-TT-lux pR-RBS_x_-lacZα-ccdB-TT*. (B–D) The mechanism of an artificial quorum-sensing circuit is the following: (B) When IPTG is added to a culture, the promoter *P_laco-1_* will trigger *luxI* and *luxR* expression, producing synthase LuxI (which produces the signaling molecule AHL) and an intracellular regulatory protein, LuxR, respectively. (C) AHLs will accumulate within and outside cells, and at or above a certain threshold, AHLs will effectively bind to LuxR. The LuxR:AHL complex will subsequently bind to the promoter *lux pR* and induce downstream expression of the lethal gene, *ccdB*, to produce killer protein CcdB. (D) Because more and more killer protein CcdB accumulates in the environment to kill large numbers of cells, the cell population density will decrease.

To build a functional system, a construct was made using multiple BioBrick gene components, which included *luxI*, *luxR*, and RBS sequences having different binding strengths (*RBS_0.07_*, *RBS_0.3_*, *RBS_0.6_*, or *RBS_1.0_*). To investigate the control of bacterial population density using a bacterial quorum-sensing system, a lethal fusion gene, *lacZa-ccdB*, driven by the promoter *lux pR* was cloned into the same construct. Furthermore, four types of RBSs with different binding strengths were assembled separately, upstream of *ccdB* to subtly control its expression strength. In addition, the promoter for the original vector, *pSB1A2*, was replaced with an IPTG-inducible promoter, *P_laco-1_*, so that the expression of the construct genes could be switched on or off as required. The essential components and mechanisms of this artificial quorum-sensing circuit are shown in Figure 1. The essential components are *P_lacO-1_-RBS_1.0_-luxI-TT-P_lacO-1_-RBS_1.0_-luxR-TT-lux pR-RBS_x_-lacZα-ccdB-TT*. The four panels of Figure 1 illustrate the regulation mechanism of each stage of the quorum-sensing process. Each panel indicates a regulation stage. For example, Figure 1A represents the cell state before the inducer IPTG is added to a culture, during which the circuit is switched off. Figure 1B–1D represent the cell states after the inducer IPTG is added to a culture, when the circuit is switched on. Figure 1B indicates that the promoter *P_laco-1_* triggers *luxI* and *luxR* expression, producing LuxI and LuxR. LuxI produces the signaling molecule AHL. Figure 1C indicates that when the concentration of AHLs reaches a certain threshold, AHLs will effectively bind to LuxR. The LuxR:AHL complex will subsequently bind to the promoter *lux pR* and induce *ccdB* expression to produce killer protein CcdB. Figure 1D indicates that because more and more CcdB accumulates in the environment to kill large numbers of cells, cell population density will decrease. Plasmids used for expressing *luxI*, *luxR*, and *ccdB* corresponding to different RBS binding strengths were successfully generated. Positive clones were confirmed by digestion with restriction enzymes and verified by Sanger sequencing (data not shown).

### Bacterial population density control by different RBS artificial quorum-sensing circuits

**Figure 2:**
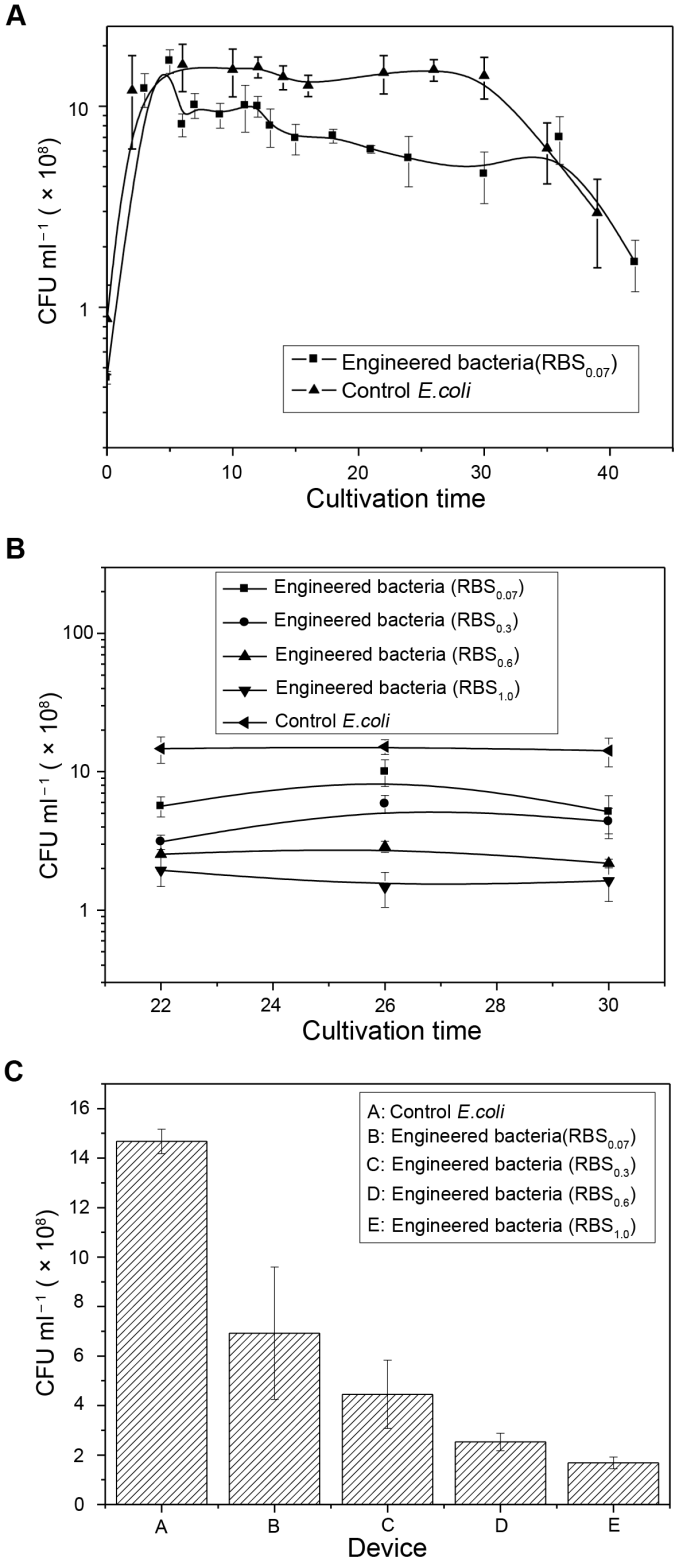
Bacterial population density controlled by different RBS artificial quorum-sensing circuits. Note: The error bars represent the standard deviation of at least three replicates. (A) Curves for viable cell numbers of the engineered bacteria *E. coli* BL21(DE3) containing an *RBS_0.07_* programmed bacterial death quorum-sensing circuit and control *E. coli*. The curve for engineered bacteria was different from that for control *E. coli*. And these differences were significant (t = 1.98 > t_0.90, 17_ = 1.74) during 6–30 h with 90% confidence interval using an independent samples t-test. At approximately 5 h, there was a dramatic decline in the number of viable engineered cells compared with control cells, after which the growth curve tended to level off after two small amplitude oscillations. The lower population density of the *RBS_0.07_* engineered bacteria remained until its growth rate decreased after >30 h of culture. (B) Viable cell numbers at different times of steady state for four strains of engineered bacteria containing different RBS (*RBS_0.07_*, *RBS_0.3_*, *RBS_0.6_*, or *RBS_1.0_*) quorum-sensing circuits. (C) Final histogram for viable cell numbers of the four strains of engineered bacteria. This indicated that with an increase in RBS efficiency, viable cell numbers gradually decreased.

Different RBS sequences upstream of the lethal gene will influence its expression which has a direct effect on the performances of standardized programmed bacterial death quorum-sensing circuits. Therefore, we constructed four types of RBS artificial quorum-sensing circuits and assessed cell growth kinetics to investigate the effect of different types of circuits on controlling bacterial cell growth and manipulating bacterial population density. These results indicated that an *RBS_0.07_*-expressing circuit could maintain a lower population density than the control bacteria (Figure 2A). This could be achieved by slowing bacterial growth or by coordinating the ratio between cell growth and death so that population density could be controlled. In addition, we chose to determine population density of bacterial growth to 22, 26, and 30 h, when bacteria had been at steady state, to indicate the control effect of engineered bacteria containing different RBS (*RBS_0.07_*, *RBS_0.3_*, *RBS_0.6_*, or *RBS_1.0_*) quorum-sensing circuits on population density (Figure 2B). In order to more intuitively show the relationship between RBS efficiency and bacterial population density, we took the average values of viable cell numbers of the four strains of engineered bacteria from Figure 2B to prepare the histogram (Figure 2C). Both Figure 2B and Figure 2C indicated that with an increase in RBS efficiency, viable cell numbers gradually decreased. Thus, the constructed *RBS_0.07_*, *RBS_0.3_*, *RBS_0.6_*, and *RBS_1.0_* artificial quorum-sensing circuits could successfully control bacterial population density at different levels. In brief, the results verified the effectiveness of the proposed mechanism of the artificial quorum-sensing circuits and the circuits were functional.

### Mathematical model and modeling analysis

The major purpose of the present study was to determine the roles and functions of the different elements in our system. In brief, the study focused on how it maintains its population density at a reasonable level by producing substances, such as killer protein, LuxI, and LuxR, and how these different proteins function together in the complex process. To further optimize this system, we applied a mathematical model to monitor the performance of our system and to analyze and predict the growth rules for bacteria with a programmed cell-death circuit. This was derived as follows.

As developed by You et al. [9], a model is
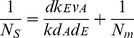
(1)


The variables and parameters in equation (1) are

*N_S_*: viable cell density at steady state (ml^−1^);

*N_m_*: cell carrying capacity in limited medium without a cell-death circuit (ml^−1^);

*d_A_*: degradation rate constant for AHL (h^−1^);

*d_E_*: degradation rate constant for killer protein (h^−1^);

*k*: cell growth rate (h^−1^);

*v_A_*: production rate constant for AHL (nM ml h^−1^);

*k_E_*: production rate constant for killer protein (h^−1^);

*d*: death rate constant for circuit-regulated cells (nM^−1^ h^−1^).

**Figure 3:**
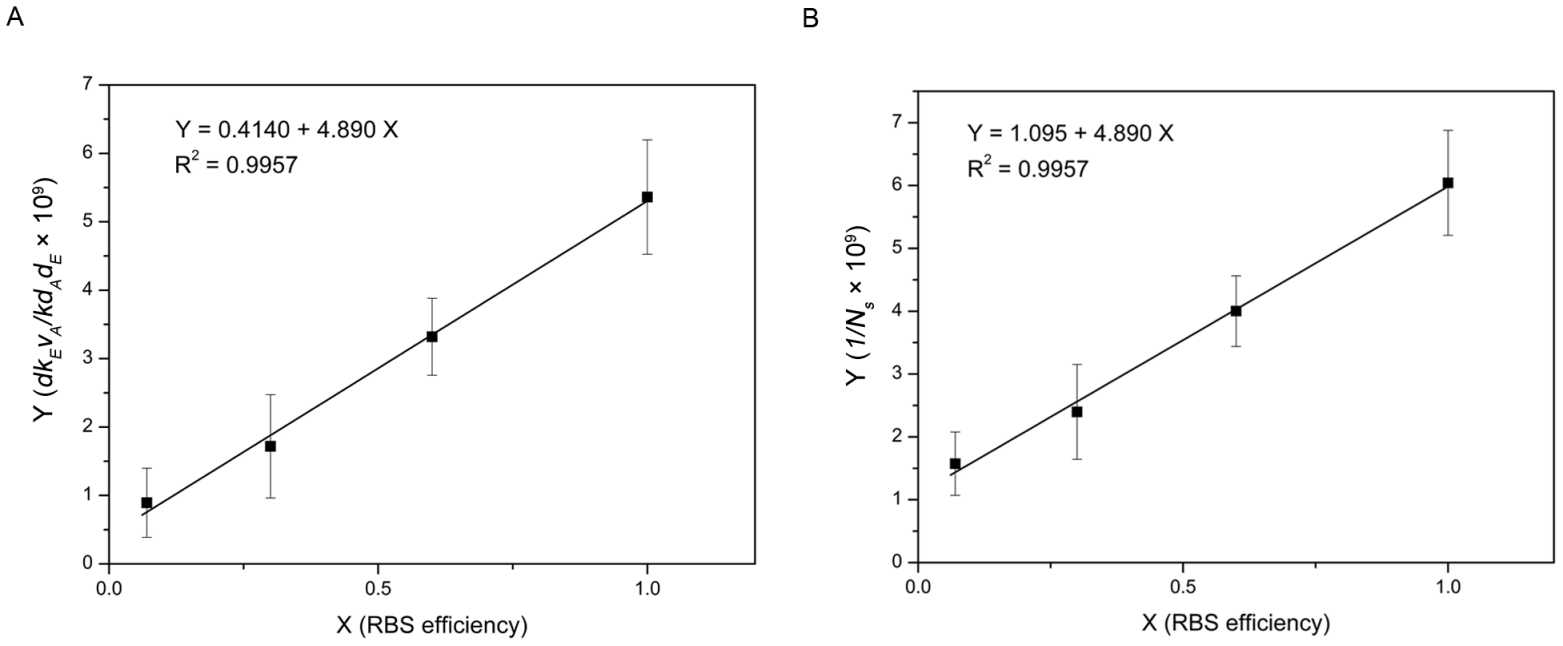
Linear relationship between RBS efficiency and model parameters. Note: *N_s_* is *the viable cell density of engineered bacteria at steady state*. The error bars represent the standard deviation of at least three replicates. (A) RBS efficiency (X) showed a linear relationship with the combined parameter 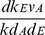 (Y): Y = 0.4140+4.890X; R^2^ = 0.9957. (B) RBS efficiency (X) also had a linear relationship with the parameter 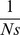 (Y): Y = 1.095+4.890X; R^2^ = 0.9957.

To characterize and compare the performances of different components of our constructed circuits, we determined cell density at steady state along with the expression of different components, which were considered to be involved in controlling bacterial population density. Based on equation (1), we observed that ***the value*** in 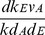 ***for*** **RBS_0.07_, RBS_0.3_,** ***and*** **RBS_0.6_** circuits (Figure 2B, 2C) was 0.14, 0.30, 0.62, while the value for the **RBS_1.0_** circuit was defined as 1.00 (Table 1). Through computational analysis using Origin 7.0 software, a linear relationship was observed between RBS efficiency (X) and 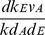 (Y), as shown in Figure 3A (Y = 0.4140+4.890X; R^2^ = 0.9957).

**Table 1:** Quantitative relationship between RBS efficiency of a bacterial population control circuit and steady-state cell density.

*N_S_*	RBS efficiency	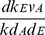
6.93×10^8^	0.07	0.14
4.45×10^8^	0.3	0.30
2.53×10^8^	0.6	0.62
1.68×10^8^	1.0	1.00

Based on these results, we derived the mathematical model shown in equation (2) to describe the relationship between ***viable cell density at steady state (N_S_)*** and RBS efficiency for *ccdB* (D). 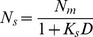
(2)


*K_S_* is a constant for how much RBS efficiency affects viable cell density. The reciprocal of equation (2) is
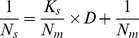
(3)


Based on the data (Table 1) for ***viable cell density of engineered bacteria at steady state*** and RBS efficiency, the linear relationship between RBS efficiency (D, i.e., X) and 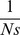 (Y) is shown in Figure 3B (Y = 1.095+4.890X; R^2^ = 0.9957).

According to our mathematical model, we predicted that when bacteria were growing ***at steady state***, a *Ks* of 4.47 and 1/*N_m_* of 1.095 would give *N_m_* of 0.91×10^9^, which was close to the experimentally measured *N_m_* of 1.47×10^9^. Therefore, the mathematical model given here reflected the relationship between RBS efficiency and ***viable cell density of engineered bacteria at steady state***. Thus, this model could be used to predict ***viable cell density of engineered bacteria at steady state*** through RBS efficiency values of a synthetic circuit.

## Discussion

Our work highlighted constructing several standardized programmed bacterial cell-death quorum-sensing circuits by cloning a series of BioBrick genes and splicing them with different RBS forms along with a lethal fusion gene, *lacZa-ccdB* using standard assembly methods. Compared with the reported research [9], the outstanding of our work is that the constructed *RBS_0.07_*, *RBS_0.3_*, *RBS_0.6_*, and *RBS_1.0_* artificial quorum-sensing circuits functioned successfully to subtly control bacterial population density at different levels by regulating *ccdB* expression.

To monitor and validate the performance of our system, we developed a mathematical model on the basis of the model of You et al. [9]. According to our results, there was a strong linear relationship between the reciprocal of ***viable cell density at steady state*** and RBS efficiency for *ccdB*. Thus, ***viable cell density at steady state could be*** quantitatively controlled by interchanging RBS sequences of different strengths in a population density control circuit. In addition, this model could predict the roles of the artificially constructed components in our system for regulating population density. For example, on account of this model, by determining the dynamics of population density, expression levels, and coordination of *luxR*, *luxI*, and *ccdB* in this system, we will know if the artificially constructed components are functioning normally and which parts need to be improved. Our system would be better optimized if the regulation levels of RBSs could be standardized so that their functions and roles could be more precisely predicted and validated.

Moreover, our current investigation systematically studied the functions of different components in regulating bacterial population density through quorum-sensing circuits. Therefore, our system provides an example and model for modulation of cell density in other field, such as fermentation industry, which requires to maintain bacterial growth at a desired density for a long time or to prolong the stationary phase of bacterial growth, so that the production of bacterial metabolites can be achieved.

Synthetic biology holds promise for developing artificial systems to address challenges posed by major global problems [33], [34] such as health [35], [36], environment [37]–[40], materials [41], and energy [42], [43]. It is advancing from the development of proof-of-concept designs to a focus on core platforms, including DNA construction, parts libraries, computational design tools, and interfaces for manipulating and probing synthetic circuits [18]. We generated a series of engineered *E. coli* cells by cloning all the essential components of a quorum-sensing circuit into the same plasmid of each engineered *E. coli* cell using the BioBrick standard assembly method. As a result, *luxR* and *luxI* expression levels in this system were regulated and coordinated by the promoter *P_lacO-1_*. Since it is an artificial simplified system, it can be an ideal platform to investigate how the quorum-sensing process works, such as by exploring its molecular signal pathway.

Furthermore, the quorum-sensing process involves in well controlled regulations and responses, so that the sensor circuit can monitor subtle change of environmental stimuli to control its own population density. Therefore, it can be potentially used as a testing or alarming system to sense environmental change, such as pollution, or microbes outbreak.
